# The role of public health information in assistance to populations living in opposition and contested areas of Syria, 2012–2014

**DOI:** 10.1186/s13031-017-0134-9

**Published:** 2017-12-22

**Authors:** Emma Diggle, Wilhelmina Welsch, Richard Sullivan, Gerbrand Alkema, Abdihamid Warsame, Mais Wafai, Mohammed Jasem, Abdulkarim Ekzayez, Rachael Cummings, Preeti Patel

**Affiliations:** 1 Save the Children, London, UK; 2 Office of the High Commissioner for Human Rights, London, UK; 3 Cancer Policy and Global Health, King’s Health Partners, King’s College London, London, UK; 4 World Vision UK, Middleton, UK; 5 Assistance Coordination Unit, Gaziantep, Turkey; 6 Save the Children Syria Response, Antakya, Hatay Turkey; 7 Global Health and Security, Department of War Studies, King’s College London, London, UK

**Keywords:** Syria, Conflict, Data, Health, Humanitarian, Mortality, Non-communicable diseases

## Abstract

**Background:**

The Syrian armed conflict is the worst humanitarian tragedy this century. With approximately 470,000 deaths and more than 13 million people displaced, the conflict continues to have a devastating impact on the health system and health outcomes within the country. Hundreds of international and national non-governmental organisations, as well as United Nations agencies have responded to the humanitarian crisis in Syria. While there has been significant attention on the challenges of meeting health needs of Syrian refugees in neighbouring countries such as Jordan, Lebanon and Turkey, very little has been documented about the humanitarian challenges within Syria, between 2013 and 2014 when non-governmental organisations operated in Syria with very little United Nations support or leadership, particularly around obtaining information to guide health responses in Syria.

**Methods:**

In this study, we draw on our operational experience in Syria and analyse data collected for the humanitarian health response in contested and opposition-held areas of Syria in 2013–4 from Turkey, where the largest humanitarian operation for Syria was based. This is combined with academic literature and material from open-access reports.

**Results:**

Humanitarian needs have consistently been most acute in contested and opposition-held areas of Syria due to break-down of Government of Syria services and intense warfare. Humanitarian organisations had to establish de novo data collection systems independent of the Government of Syria to provide essential services in opposition-held and contested areas of Syria. The use of technology such as social media was vital to facilitating remote data collection in Syria as many humanitarian agencies operated with a limited operational visibility given chronic levels of insecurity. Mortality data have been highly politicized and extremely difficult to verify, particularly in areas highly affected by the conflict, with shifting frontlines, populations, and allegiances.

**Conclusions:**

More investment in data collection and use, technological investment in the use of M- and E-health, capacity building and strong technical and independent leadership should be a key priority for the humanitarian health response in Syria and other emergencies. Much more attention should be also given for the treatment gap for non-communicable diseases including mental disorders.

## Background

The Syrian armed conflict which began in 2011 is the deadliest of recent wars and the worst humanitarian catastrophe this century. Estimates suggest that more than 470,000 people have died according to a report pubished by the Syrian Centre for Policy Research in February 2016 – this number has likely to have increased although there are no recent reports [1]. More than 13 out of 23 million Syrians have been displaced both internally and externally [2–4]. Widespread crimes against humanity have been committed by Government of Syria (GoS) and opposition forces [5, 6]. Control of the country is currently divided among the GoS, Islamic State (ISIS), Kurdish forces and a variety of armed opposition forces [7, 8].

Syria has become one of the most dangerous places for healthcare providers – a strategy described as the weaponisation of healthcare in a recent study has meant that hundreds of healthcare workers have been killed and/or tortured, and several health facilities deliberately destroyed [9]. As a result, Syria’s health system has been impacted catastrophically, with supply lines interrupted, and a general degradation of key services [10]. The economic cost of the conflict is estimated to be £175bn ($255bn). The Syrian conflict continues to have dire socio-economic consequences with 82.5% of Syrians living in poverty and life expectancy having decreased from 70 years (2010) to 55.4 years (2015) [1].

Hundreds of international and national non-governmental organisations (NGOs), as well as United Nations (UN) agencies are responding to this crisis. The humanitarian response for inside Syria has been largely managed either from within Syria or from neighbouring countries, particularly Turkey. Needs have consistently been most acute in contested and opposition-held areas of Syria due to breakdown of GoS services and intense warfare. The delivery of humanitarian aid to these areas has been severely hindered by insecurity, the GoS (which prohibited most non-UN actors from operating legally) and lack of leadership from the UN. As a result, many international NGOs have had to operate remotely through local implementing partners [11]. In 2014, the UN finally authorised its agencies to use named border crossings into Syria for humanitarian aid without consent from the GoS [12, 13]. This enabled the World Health Organisation (WHO) and other UN agencies to be more visible and engaged throughout Syria. However, between 2013 and 2014 NGOs operated in Syria with very little UN support or leadership, and very little is known about the challenges of the humanitarian health response during this time.

During conflict, data on mortality, morbidity and health services are essential for establishing needs, designing and evaluating humanitarian interventions and documenting health impacts on civilians for historical but also real-time advocacy uses with donors and parties to the conflict. Such data should also be collected to support timely and appropriate public health action [14, 15]. To date, most data on war-affected Syrians have been generated among refugee populations in Lebanon, Jordan and Turkey [16–37]. Little substantive public health analysis has been published for populations living inside Syria, particularly those in contested and opposition-held areas [38, 39]. For example, a Pubmed search showed only seven publications on health inside Syria since the war began [9, 39–45]. Generally, there is substantially greater evidence on health needs of refugees than those of populations trapped within conflict-affected countries.

### Context of data collection

Up until the end of 2014, NGOs operated in contested and opposition-held areas from neighbouring countries with very limited UN guidance and support, and no formal coordination mechanism. Endorsed by reform of the humanitarian system in 2005, the Cluster Approach aims to improve coordination, leadership and accountability of different humanitarian sectors such as health, nutrition, protection, and logistics. Clusters are partnerships of humanitarian organizations, both UN and non-UN [46]. In 2013–2014, clusters had not formally been activated by the UN for the cross-border response, despite Syria being classified as level three (i.e. highest) in the UN emergency grading. This failure to institute standard coordination mechanisms and the very limited presence of UN agencies, who lead most humanitarian clusters, in opposition and contested-areas of Syria, has been criticised elsewhere [47]. In particular, the World Health Organization (WHO)‘s close working relationship with GoS Ministry of Health (MoH), and conflicting mandates of agency coordination as well as host government assistance, meant that it was not directly involved in coordination mechanisms for the humanitarian health response led from Turkey, and did not undertake or oversee vital public health information activities [47–49]. Instead, national and international NGOs set up a Health Working Group (HWG; now known as the NGO Forum for Non-Governmental Organisations Operating in northern Syria) in mid-2013 to provide coordination and information exchange for cross-border humanitarian health interventions from southern Turkey.

The HWG attempted to replicate typical health cluster ways of working, and engaged with the Interim Ministry of Health set up by the Syrian opposition. Many agencies operated with a limited presence and visibility, partly due to delays in obtaining formal registration from Turkish authorities. In terms of public health data, the HWG had to establish de novo data collection systems independent of those in GoS areas, since there were no formal open channels of communication with the actors working in GoS controlled areas. Most data collection activities reviewed in this study were undertaken by the HWG.

This paper reviews the collection, analysis and use of public health data in contested and opposition-held areas of Syria in 2013 and 2014, as a major case study of challenges in such contexts, and recommends improvements based on the Syrian experience. When considering different public health information needs, we broadly follow the framework set out in a recent study by Checchi et al. [50].

## Methods

The study draws on the multiple operational experiences of the authors as humanitarian health professionals working in the Health Working Group (NGOs based in Turkey), the Syrian Strategic Needs Analysis Project (SNAP) (which supports the humanitarian response in Syria by providing independent analysis and supporting coordinated assessments), and the Assistance Coordination Unit (an NGO coordination body which set up the Early Warning Alert and Response Network (EWARN). More specifically, ED was the Public Health Information Manager with the Health Working Group in Turkey; WW was the lead analyst for SNAP and an assessment coordinator for the food security cluster in Gaziantep, Turkey; RS is a non-communicable diseases and health policy expert; GA has worked in Turkey to support the Syrian Immunisation Task Force; AW is a public health information specialist and has worked in many large scale humanitarian health crises; MW is a Syrian paediatrician working with EWARN and managing its nutrition component; MJ is a Syrian medical expert and manages EWARN; AK is a Syrian medical doctor and epidemiologist who led the health response in north-west Syria with Save the Children; RC is senior humanitarian health advisor at Save the Children; and PP is a social scientist conducting research and capacity-building work in conflict-affected countries.

We also reviewed data from publically available reports such as Multi-Sector Initial Rapid Assessments (MIRA), Dynamic Monitoring System (DYNAMO), Joint Rapid Assessments in northern Syria (JRANS) and individual non-governmental NGOs. This was supplemented with academic and grey literature from PubMed, Reliefweb and Google using search terms such as Syria, conflict, war, health, health information, humanitarian, data, diseases and health system.

## Results

### Affected population size and general needs assessment

In contested and opposition-held areas of Syria throughout 2013–4, the United Nations Office for the Coordination of Humanitarian Affairs (UNOCHA) produced administrative district profiles on a bi-annual basis without specifying any of the methods used. Information about population size was also collected by humanitarian agencies through consecutive Multi-Sector Initial Rapid Assessments (MIRA), coordinated by UNOCHA and designed to identify priorities during the first weeks following an emergency [51–53]. These very lengthy assessments (1109 variables of which 149 were for health) relied on a mix of primary and secondary data, the former collected by Syrian interviewers supervised remotely and consisting of structured questionnaires, community information interviews and focus groups selected through convenience sampling. To minimise risk for interviewers and respondents, recording of responses was minimised and instead, qualitative data were transcribed during extensive debriefing of interviewers. Among other topics, the MIRA explored population displacement dynamics [54].

In addition to MIRA, a more abridged, multi-sector needs monitoring exercise (DYNAMO) was carried out from March 2014 with irregular (every 2–5 months) frequency by the Assistance Coordination Unit, a Syrian NGO umbrella body based in Turkey, in areas it accessed [55]. DYNAMO was also remotely implemented and reliant on convenience sampling, though emphasis was placed on validating and cross-checking information on attacks and major events experienced by the population [56].

The main limitations of MIRA and DYNAMO were that they initially sought to collect an unwieldy amount of information, resulting in unclear quality of responses, long delays (particularly for MIRA) in implementation, analysis and publication, and difficulty in longitudinal comparisons due to questionnaire changes. These were, however, the main instruments available to obtain a comprehensive insight into the needs of people inside opposition-held and contested areas of Syria.

### Public health risks

#### Exposure to armed attacks

Exposure to attacks can be recorded through different methods such as population surveys, media and human rights activist reports, hospital records, and capture-recapture statistics [57]. In addition, the humanitarian protection cluster has developed standardized reporting forms that feed into an event-based surveillance system. In the humanitarian response led from Turkey in 2013–4, such data were collected predominantly through social media reports, particularly as they concerned assaults on hospitals, doctors, and patients [58, 59]. In 2014, for example, Physicians for Human Rights documented 224 attacks on medical facilities and 600 deaths of medical personnel in Syria since the start of the conflict [60, 61]. Violations against human rights and international humanitarian law, including cases of attacks against medical personnel and facilities, injuries and sexual and gender-based violence, were at times shared with the protection sector or human rights actors. However, accurate estimates of population exposure to armed attacks were not obtained, as these would have necessitated more detailed methods such as self-reporting household sample surveys [62]. Most agencies had limited capacity to carry out such surveys, and furthermore they would have entailed data collection in extremely insecure conditions, with limited provisions for minimising the possible untoward effects for data collectors or respondents of collecting or sharing sensitive information on attacks.

#### Disease burden: Proportional morbidity

Data on proportional morbidity in health facilities are typically available via a Health Information System (HIS). In contested and opposition-held areas of Syria in 2013–4 such a system was neither systematic nor standardised. Different NGOs set up individual data collection systems for their own programmatic needs, often collecting data that had not been defined or compiled using uniform standards and definitions. This made reporting, monitoring and evaluation difficult. No overall HIS reporting was set up largely due to lack of capacity and leadership in the Health Working Group. Although considerable time and effort was devoted to developing the use of a shared, web-based open-source health information system application DHIS-2 (https://www.dhis2.org), lack of translation into Arabic curtailed its usability inside Syria [63].

#### Disease burden: Epidemic occurrence

In order to detect epidemic risk and occurrence*,* the Assistance Coordination Unit (ACU) a NGO Coordination body based in Turkey and belonging to the humanitarian section of Syrian National Coalition set up an Early Warning Alert and Response Network (EWARN) system in 2013, following the WHO EWARN Guidelines [64]. At the end of 2014, the ACU EWARN covered a reported population of 10,003,000, reporting from 321 out of a possible 335 sentinel health facilities in nine of the 14 Syrian governorates; and had generated 3502 alerts (see Table 1). Confirmed disease outbreaks identified by the ACU EWARN since 2013 included poliomyelitis, acute jaundice syndrome, typhoid and measles. Data were published on social media and the web (http://www.acu-sy.org/en/) [65, 66].

**Table 1:** Summary of epidemic alerts generated by the ACU EWARN in 2014

Syndrome	Number of alerts
Acute bloody diarrhoea	125
Acute watery diarrhoea	0
Acute jaundice syndrome	642
Severe acute respiratory infection	113
Acute flaccid paralysis	65
Measles	1003
Meningitis	20
Unexplained cluster of events	101
Unexplained death	45
Fever of unknown origin	344
Leishmaniasis	74
Suspected typhoid fever	970

The Syrian MoH, supported by WHO Syria, also operated a disease Early Warning System (called EWARS) which started in September 2012, reporting in 104 out of a possible 650 sentinel facilities in 14 Syrian governorates by late 2014 (Table 1) [67]. Data were also analysed on a weekly basis, and published online [68]. Both EWARN and EWARS were simplified disease surveillance systems set up in response to the conflict, monitoring a limited number of priority epidemic-prone diseases using syndromic definitions, such as acute jaundice, acute diarrhoea and measles. The failure to harmonise these surveillance systems due to lack of coordination between contested and opposition-held areas of Syria, versus Government of Syria controlled areas inevitably meant that case ascertainment and reporting were being duplicated in 2014, although we do not know to what extent this was the case. In addition, case definitions and alerts were not shared between the two systems. There were also differences in diseases covered; for example, cutaneous leishmaniasis was not reported by the MoH EWARS, but was added to EWARN following reports of much-increased incidence (Syria is one of the most affected countries, and Aleppo the most highly endemic city worldwide) [69, 70].

Despite regular and timely reporting, the ACU EWARN lacked resources and authority to investigate and respond to alerts generated by the system, especially in areas where neither the Syrian MoH nor the Interim Government MoH were functioning. Thus, alarming epidemic alerts, such as large numbers of measles cases, were not followed by an appropriate public health response. The EWARN announced an outbreak of Measles since the 4th week of 2014, reporting between 100 and 150 cases of measles weekly (in the week 29/2014 there were 145 cases of measles were reported by EWARN), and yet there was no effective response as EWARN lacked capacity. Other diseases such as acute bronchiolitis, whooping cough, acute diarrhoea, brucellosis, cutaneous leishmaniasis and typhoid were being reported and also lacked an appropriate response due to lack of ACU capacity. This meant that results were not used in a timely manner despite having set up a comprehensive system at the outset of the conflict. Inorder to maximise usability of data in an extraordinatorily complex system as described here, the ACU and similar organisations should perhaps have been supported by an entirely independent specialised agency with technical expertise to respond to such epidemics most effectively.

#### Disease burden: Non-communicable diseases and mental disorders

Before the conflict, some studies suggest that adults in Syria had the highest prevalence of cardiovascular disease risk factors in the world, with 45.6% for hypertension, 43.2% for obesity, 21.9% for hypercholesterolemia and 15.6% for diabetes [71–73]. Despite these alarming trends, reliable surveillance of cardiovascular disease and its risk factors was absent in Syria suggesting weak baseline data. The Syrian Center for Tobacco Studies, had begun efforts to provide the first comprehensive assessment of the spread and distribution of cardiovascular disease risk factors in Syria but this was stymied by the conflict [71].

Reports suggest that there have been a projected 300,000 deaths due to chronic non-communicable diseases (NCDs) in Syria since the beginning of the conflict, due to widespread discontinuation of treatment [3]. Data on the burden of NCDs and the extent of unmet treatment need were not collected systematically through a Health Information System, nor to our knowledge was there an attempt to identify and register patients in need of treatment continuation, as recommended [57].

Information about mental disorders and services should be collected through a desk review of available reports, including pre-war burden estimates, supplemented by participatory assessments, and data gathered through general health assessments and Health Resources and Services Availability Mapping System (HeRAMS) (see below) [57]. In contested and opposition-held areas of Syria, there were no systematic efforts to compose a general picture of the burden of mental disorders, partly due to limited capacity to provide services both before and during the conflict [74, 75]. Both the 2014 HeRAMS and 2009 Syrian Household Survey were not specific in terms of mental disorder categorization.

We attempted to illuminate the importance of the chronic disease information gap by estimating the percentage and number of NCD and mental disorder cases not receiving treatment (treatment gap) as of October 2014 (Table 2). To do this, we projected caseload by multiplying the available population figures for contested and opposition-areas of Syria by pre-war estimates of the prevalence of the main NCDs (diabetes, hypertension) and mental disorders [51]. We present two scenarios, based on a 2009 Syria-wide survey and regional estimates, respectively (see sources in Table 2). We roughly approximated access to treatment using as a proxy the percentage of health facilities within this population that reported functionality of services to manage NCDs and mental disorders, as collected by a Health Resources and Services Availability Mapping System (HeRAMS) survey [76] (see below). We multiplied this percentage by the projected caseload to compute the number receiving treatment. Taking a cautious approach, these estimates assume that the war did not result in an increased prevalence (in reality, there is strong evidence that the prevalence of mental disorders such as depression and particularly post-traumatic stress disorder (PTSD) increase as a result of war conditions [77, 78]), and are subject to further bias resulting from the incomplete coverage of HeRAMS data collection (see below), and the possible disconnect between availability of services and actual access to care, e.g. due to insecurity. Nevertheless, our estimates suggest a large treatment gap in contested and opposition-held areas of Syria, with most cases going without treatment, as opposed to the 14% gap in continuous treatment estimated for 2009.

**Table 2:** Estimated treatment gap for common NCDs and mental disorders in, 2013

Disease	Prevalence estimate (source)	In need of treatment	Receiving treatment	Treatment gap (%)
Diabetes^a^	1.8% (Syrian National Household Survey, 2009)	281,000	45,000	236,000 (84%)
	12.1% (regional average, 2014)	1,872,000	300,000	1,573,000 (84%)
Hypertension^b^	2.3% (Syrian National Household Survey, 2009)	359,000	25,000	327,000 (91%)
	41% (regional average, 2010)	3,511,000 (6,396,920)	316,000 (575,723)	3,195,000 (5,821,197) (91%)
Depression^c^	0.4% (Syrian National Household Survey, 2009)	62,000	47,000	16,000 (75%)
	(14.95%) (regional average, 2011)	(2,332,535)	1,749,402	583,134 (75%)

Information sources:

^a^Diabetes- regional average for 2014 [104–106]

^b^Hypertension- regional average for 2010 for data from Turkey, Egypt, Saudi Arabia [106, 107]

^c^Depression - an average of Lebanon, Egypt, Pakistan, United Arab Emirates, Iran, Iraq [108]

To our knowledge no such projections of treatment gap were made in real-time during 2013–2014, despite the availability of pre-conflict prevalence data; this should become an essential health information exercise, directly informing the public health response [24, 79]

While we present data on the estimated NCD and mental health burdens, we acknowledge that we do not present data on key maternal health outcomes such as maternal and neonatal mortality. The inclusion of the former are intended as examples and future data should include analysis of other critical health outcomes such as reproductive health, and key health risk factors such as access to safe water supplies and sanitation.

##### Public health services

The Health Resources and Services Availability Mapping System (HeRAMS) is a standardized approach supported by a software-based platform that aims to strengthen the collection, collation and analysis of information on the availability of health resources and services in humanitarian emergencies [80]. HeRAMS surveys all health facilities and assesses their functionality status, accessibility, health infrastructure, human resources, availability of different health services, equipment, and medicines at primary and secondary care level. A Syria-wide HeRAMS survey was initiated by the Syrian MoH and WHO in early 2013. Results, however, were only made available in April 2014 [81]. The survey showed that the conflict had resulted in enormous losses of health staff, and severely reduced medical supply routes. It also suggested that 43% of accessible public hospitals and 21% of accessible public primary health facilities were completely or partially damaged, resulting in areas with no access to health care [76, 82].

This first HeRAMS exercise was of limited use for agencies working in contested and opposition-held areas of Syria, since only governorate-level aggregate results were made available: data for individual health facilities were not shared, and the survey’s coverage in contested and opposition-held areas was suspected to be low [83]. Thus, a separate HeRAMS was initiated by the HWG in Turkey in July and August 2015, with data for opposition-held and contested areas of Syria on the availability of basic health services (Table 3) [84]. This exercise was rendered arduous by the need to establish a de novo database of health facilities, given no pre-conflict health system data were available from the Syrian MoH.

As a less data-intensive, more frequently updated alternative to HeRAMS, the HWG maintained a 4 W (Who is doing What, Where and When) database, including broad information on each agency’s areas of service delivery (e.g. reproductive health, surgery, etc.). However, strict information sharing protocols dictated by security concerns (e.g. possible targeting by combatants if agencies’ locations of operation were disclosed) meant that only aggregated results for each governorate could be published, with no public detail on what each agency was doing where. This constrained identification and response to priority geographic and service area gaps in service provision. For example, during the preparations for both the measles and polio vaccination campaigns in 2014, UN agencies, cross-border NGOs, the Syrian MoH in Damascus, NGOs working in GoS areas, and the interim MoH in opposition-held and contested areas all conducted largely overlapping micro-planning for the campaigns, based on information available to them [85]. Multiple vaccination of the same children was commonly reported. Independent campaign monitoring was performed by the Qatari Red Crescent, but agencies responsible for vaccination were not given access to monitoring data for their operational areas. Vaccination coverage was estimated by the ACU using the administrative method [86], which is commonly biased by over-reporting of vaccination outputs and inaccuracy in target population denominators [57].

With the exception of vaccination campaigns, we are not aware of other systematic efforts to quantify utilisation of health services in contested and opposition-held areas of Syria, for which the Sphere Guidelines specify clear standards [87]. While different agencies were collecting various coverage and utilisation data in their catchment areas, a uniform Health Information System, combined with population figures, would have provided an overall picture of service utilisation, indicating the actual extent of health care access.

##### Population mortality

In contested and opposition-held areas of Syria, opportunities to collect all-cause population mortality data through joint rapid needs assessments were tested. Specifically, assessment teams asked key informants (e.g. community leaders) to compile death counts from available sources, including Syrian Arab Red Crescent lists, hospital data, local council records and lists provided by combatants. Mortality and injury counts provided by key informants in contested and opposition-held areas of Syria between January and April 2013 recorded a total of 144,272 deaths and injuries (Table 4). Mortality data were not collected in this way after April 2013, as they were not considered a priority for decision-making. Standard mortality surveys were also not carried out, and judged unfeasible for security reasons.

**Table 3:** Availability of basic health services in % of facilities offering the service, by level of healthcare, July–August 2015

Level of health care / Type of health service	Primary health care	Secondary health care	Tertiary health care	Total
Outreach activities within the community				
Health education	43.6	18.9	43.2	37.8
Screening for malnutrition with MUAC	27.5	18.9	13.6	23.2
Follow up of malnourished children	30.4	39.6	18.2	30.2
Pregnancy screening for referral to ANC	36.9	39.6	27.3	35.8
Screening and referral of non-vaccinated children	28.2	28.3	11.4	24.8
Out Patient services				
Outpatient services	63.1	83	70.5	68.7
Basic Laboratory Services	30.2	84.9	47.7	45.1
Basic Imaging Service	15.4	79.2	50	35.4
Surgery				
Primary Injury care	52.3	86.8	38.6	57.3
Emergency surgery	17.4	73.6	43.2	34.1
Elective surgery	12.8	75.5	40.9	31.3
Intensive care unit	4.7	32.1	11.4	11.8
Basic blood bank service	6.7	49.1	29.5	19.9
Comprehensive blood bank service	0.7	7.5	11.4	3.7
Post-operative care	24.8	77.4	45.5	39.8
Child health				
EPI	17.2	13.2	2.3	13.6
Screening for acute malnutrition (SAM)	22.1	28.3	18.2	22.8
Outpatient treatment of SAM	24.2	45.3	22.7	28.5
Stabilization Centre for the management of Severe Acute Malnutrition	8.1	15.1	18.2	11.4
Basic Child Care (IMCI)	28.4	32.1	29.5	29.4
Management of children suffering from severe and very severe illness	37.6	57.7	34.1	41.2
Communicable diseases				
Treatment of measles	49.7	56.6	34.1	48.4
Treatment of cholera	25.5	35.8	31.8	28.9
Treatment of acute bloody diarrhoea	54.7	67.9	38.6	54.7
Treatment of acute watery diarrhoea	58.4	67.9	38.6	56.9
Treatment of Typhoid and Brucellosis	57.7	73.6	34.1	56.9
Treatment of Rabies	16.1	15.1	6.8	14.6
Treatment of leishmaniasis	73.8	56.6	31.8	62.6
Diagnosis of Viral Hepatitis B&C	33.1	52.8	31.8	37.1
Treatment of Viral Hepatitis B&C	5.4	13.2	13.6	8.5
Diagnosis of TB locally or via referral	33.6	47.2	20.5	34.1
Treatment of TB	4.7	11.3	11.4	7.3

Source: Health Resources Availability Mapping System (HeRAMS) Health Facilities Report:

Health Cluster - Turkey: Assessment of 254 facilities in nine Governorates in Syria. July–August 2015, 2015: http://reliefweb.int/sites/reliefweb.int/files/resources/herams_report_-_final.pdf

**Table 4:** Mortality and injury counts provided by key informants in contested and opposition areas of Syria, January and April 2013

		January 2013			April 2013			Grand total (dead + injured)
Variable	Governorate	Dead	Injured	Total	Dead	Injured	Total	
Children <5y old	Al-Hassakeh	6	20	26	33	287	320	346
	Aleppo	135	658	793	739	4227	4966	5759
	Ar-Raqqa	18	0	18	123	535	658	676
	Deir-ez-Zor	136	606	742	491	1857	2348	3090
	Hama	n/a	n/a	n/a	304	1535	1839	1839
	Idleb	1003	1112	2115	527	3207	3734	5849
	Lattakia	19	275	294	13	43	56	350
	Total	1317	2671	3988	2230	11,691	13,921	17,909
Female ≥5y old	Al-Hassakeh	5	15	20	36	89	125	145
	Aleppo	257	1747	2004	770	5880	6650	8654
	Ar-Raqqa	29	26	55	132	1031	1163	1218
	Deir-ez-Zor	446	1407	1853	385	1346	1731	3584
	Hama	n/a	n/a	n/a	248	1905	2153	2153
	Idleb	892	690	1582	450	1991	2441	4023
	Lattakia	33	325	358	29	25	54	412
	Total	1662	4210	5872	2050	12,267	14,317	20,189
Male ≥5y old	Al-Hassakeh	81	51	132	444	583	1027	1159
	Aleppo	1277	6201	7478	3778	19,642	23,420	30,898
	Ar-Raqqa	370	244	614	1637	2701	4338	4952
	Deir-ez-Zor	2760	18,027	20,787	3726	8107	11,833	32,620
	Hama	n/a	n/a	n/a	2879	13,736	16,615	16,615
	Idleb	2608	3209	5817	3615	7616	11,231	17,048
	Lattakia	446	1650	2096	236	550	786	2882
	Total	7542	29,382	36,924	16,315	52,935	69,250	106,174
Column grand totals		10,521	36,263	46,784	20,595	76,893	97,488	144,272

Separately, Syrian and international civil society organisations, news agencies, and the Syrian government have all collected data on people killed, mainly for advocacy and documentation, rather than public health purposes, in many cases publishing nominal lists with extensive detail on circumstances of death [88]. These disparate sources were subjected to extensive cleaning and multiple records linkage by the Human Rights Data Analysis Group, on behalf of the Offices of the United Nations High Commissioner for Human Rights (OHCHR), to generate a minimum credible number of violent deaths [89–92]. While the completeness of available registries is unknown, the extensive network of ground informants and activist organisations, a feature of the Syrian conflict, has probably contributed to documenting violent mortality in Syria more extensively than in other recent wars. However, accurate estimates of excess mortality, including deaths caused indirectly by the conflict, remain missing [93].

## Discussion: Key lessons from Syria

### Information sharing

Strict information sharing protocols were implemented to allay security and authorisation concerns of agencies working cross-border and from GoS-held areas. However, these agencies were reluctant to share information with WHO, for fear of leakage to the GoS. As a consequence, very little useful information was shared, severely curtailing coordination and strategic planning. Data on locations of health facilities and programmes is often used for direct targeting by combatants on health workers and health care more broadly as illustrated by attacks in Syria, Afghanistan, Yemen, and several other conflict-affected settings [94–96]. In future conflict settings, health information could be managed by an entirely independent specialised agency [97].

### Population denominators

Uncertainty about population denominators impeded meaningful analysis of health data (e.g. coverage indicators) and accurate service delivery planning (e.g. for vaccination campaigns or pharmaceutical procurement). Denominator uncertainty was due to a i) a high level of internal and external displacement and the limited capacity to monitor in- and out-flows across borders and different facility catchment areas; ii) the collapse of pre-war statistical services; iii) out-dated census figures (2004); and iv) a potential bias of key informants asked to provide population figures (inflation of figures to maximise relief rations is a known phenomenon in humanitarian settings). More investment should be made into establishing a regularly updated reference point in order to have baseline data to work from [98]. In order to have a clearer understanding of population health needs, greater investment should be made into improving the quality of essential baseline data such as population denominators.

### E-health and m-health opportunities

Most data were collected remotely, making it difficult to check reliability. Mobile messaging apps such as Whatsapp were popular forms of communication, but transferring large documents via this medium was difficult and dependent on intermittently functioning mobile networks. As collection of data via soft and hard computer files were deemed to be unsafe to due to risk of interception, data were also often collected by committing information to memory. In effect this could have led to a high risk of recall bias. It is expected that modern technology will provide an unprecedented ability to monitor, detect, and respond to crises in similar settings such as Iraq, Libya, Ukraine where social media use, cell-phone and internet connectivity is growing rapidly [14, 99]. Further investment and preparedness into solutions involving mobile phone software should be made. Field assessments can be conducted via mobile phones using free technology similar to limesurvey, and data can be relayed to servers for quick analysis and a fast response [100, 101]. Investment must continue into key e-health tools such as DHIS2 (Health Management Information Systems) and tele-reporting to ensure preparedness for use in all contexts allowing for appropriate language capacity. This will encourage and reassure professionals on the ground that it is worth utilising and discourage development of incompatible systems at field level.

### Agencies’ capacity for data collection

Throughout 2013–14, NGOs on the ground were entirely responsible for health data collection and analysis, without support from WHO. Coordination staff were very limited, and staff turnover was high, resulting in discontinuity of planning and delays between data collection and action. For example, much effort went into repeated cross-sectional needs assessments, rather than setting up functional prospective systems such as HIS. Syrian NGOs, arguably the best placed for data collection due to their capillary presence inside Syria and high motivation, faced language barriers and had limited opportunities for on-site training on health information methods.

Coordination mechanisms should be staffed with stable teams, including specialists in health information methods, backed up by technical networks for distance support, e.g. from WHO, the Centers for Disease Control and academic centres of excellence. Real-time training of agencies, particularly local entities, in methods and ethical provisions of health data collection should be a consistent function of health clusters and other coordination mechanisms.

## Conclusion

In September 2014, the Whole of Syria approach was adopted to bring together the cross-border humanitarian assistance from Turkey and Jordan into a single framework [102]. This appears to be improving efficiency and may ensure greater accountability, effectiveness and reach. Clusters have been activated in cross-border responses and the three hubs (Damascus in Syria, Amman in Jordan and Gaziantep in Turkey) are working together to ensure that health needs are jointly assessed with priorities identified, and health information is promptly shared. The UN is now more engaged, and there has been a move toward creating common data collection systems such as EWARN and HeRAMS between the Government of Syria and the contested and opposition-held areas of the country. However, the conflict continues to worsen with growing barriers to the delivery of aid, high levels of insecurity, and the fragmentation and radicalization of armed groups in many areas of Syria [103].

Reliable and timely information to support evidence based decision-making to respond to the health crisis among Syrians remains far from ideal, but has improved over time. There is no doubt that humanitarian agencies encountered many challenges in collecting health data in Syria from 2013 to 2014. These included having to maintain strict information sharing protocols, limited sharing of data, duplication of key activities, lack of leadership and coordination, limited NGO capacity and little UN support. We call for a much greater focus on health data within Syria and similar conflicts, and greater engagement by international donors to support this work. Although this paper provides a limited snapshot of the health status in Syria, it raises key points about the need for more health data within Syria and within other humanitarian crises. In future conflict-affected settings, health information could perhaps be managed by an entirely independent specialised agency, and better investment in E- and M-health should be a priority given the growing security and governance challenges in Syria and elsewhere.

### Key messages

- Between 2013 and 2014, access for humanitarian aid to contested and opposition-held areas of Syria was severely hindered by insecurity, the Government of Syria and lack of leadership from the United Nations
- Humanitarian needs have consistently been most acute in contested and opposition-held areas of Syria due to breakdown of Government of Syria services and intense warfare
- Humanitarian organisations had to establish de novo data collection systems independent of the Government of Syria to provide essential services in opposition-held and contested areas of Syria
- The use of technology such as social media was vital to facilitating remote data collection in Syria as many humanitarian agencies operated with a limited operational visibility given chronic levels of insecurity
- Mortality data have been highly politicized and extremely difficult to verify, particularly in areas highly affected by the conflict, with shifting frontlines, populations, and allegiances
- Much more attention should be given for the treatment gap for non-communicable diseases including mental disorders
- More investment in data collection and use, technological investment in the use of M and E-health, capacity building and strong technical and independent leadership should be a key priority for the humanitarian health response in Syria and other emergencies

## Abbrevations

ACU: Assistance Coordination Unit
DHIS2: District Health Information Systems 2
DYNAMO: Dynamic Monitoring System
EWARN: Early warning network
EWARS: Early warning and response system
GIS: Geographical information system
GoS: Government of Syria
HeFRA: Health facility rapid assessment
HeRAMS: Health Resources Availability and Mapping System
HIS: Health information systems
HWG: Health Working Group
IASC: Inter-Agency Standing Committee
IDPs: Internally displaced persons
INGO: International non-governmental organisation
IRA: Initial Rapid Assessment
ISIS: Islamic State
JRANS: Joint Rapid Assessments in northern Syria
MIRA: Multi-Sector Initial Rapid Assessments
MoH: Ministry of Health
MSNA: Multi Sector Needs Assessment
NCD: Non- communicable disease
NGO: Non-Governmental Organisation
OCHA: Office for the Coordination of Humanitarian Affairs
OHCHR: United Nations High Commissioner for Human Rights
SHARP: Syria Humanitarian Response Plan
SINA: Syria Integrated Needs Assessment
SNAP: Syrian Strategic Needs Analysis Project (SNAP)
SNGO: Syrian Non-Governmental Organisation
SOHR: Syrian Observatory for Human Rights
SRP: Strategic response plan
UN: United Nations
WHO: World Health Organisation
